# Recycling of Waste Cotton Textile Containing Elastane Fibers through Dissolution and Regeneration

**DOI:** 10.3390/membranes12040355

**Published:** 2022-03-24

**Authors:** Luxuan Wang, Shuting Huang, Yixiang Wang

**Affiliations:** Department of Food Science and Agricultural Chemistry, McGill University, Ste Anne de Bellevue, QC H9X 3V9, Canada; luxuan.wang@mail.mcgill.ca (L.W.); shuting.huang@mail.mcgill.ca (S.H.)

**Keywords:** waste cotton textile, elastane fiber, recycling, dissolution, regeneration

## Abstract

Increasing utilization of textiles has raised concern regarding the environmental impact brought by the textile manufacturing process and disposal of waste textiles. In our previous work, the dissolution of cotton waste through different solvent systems was demonstrated. Herein, this study aimed to further investigate the recycling of waste cotton–elastane fabrics using H_2_SO_4_, NaOH/urea, and LiCl/DMAc solvent systems. The structure of regenerated films was characterized with Fourier transform infrared spectroscopy and scanning electron microscopy, and the properties of the regenerated films, including transparency, mechanical properties, water vapor permeability, and thermal stability, were investigated. The results revealed that all solvent systems could convert the waste cotton–elastane fabrics into regenerated films with the existence of different forms of elastane components. The elastane fibers were partially hydrolyzed in H_2_SO_4_ solvent and reduced the transparency of regenerated films, but they were well retained in NaOH/urea solvent and interrupted the structure of regenerated cellulose films. It is worth noting that the elastane fibers were completely dissolved in LiCl/DMAc solvent and formed a composite structure with cellulose, leading to obviously improved tensile strength (from 51.00 to 121.63 MPa) and water barrier property (from 3.50 × 10^−7^ to 1.03 × 10^−7^ g m^−1^ h^−1^ Pa^−1^). Therefore, this work demonstrates the possibility to directly recycle waste cotton–elastane fabrics through dissolution and regeneration, and the resultant films have potential applications as packaging materials.

## 1. Introduction

Owing to the growing global population alongside the increasing demand for clothing, the textile industry has become one of the most important and biggest industries in the world [1]. In 2018, global fiber production has exceeded 105 million metric tons [1,2]. Conventional treatments of waste textile include municipal landfill and incineration. For landfills, large space is occupied, and the decomposing period can range from 6 months to 20 years depending on the fiber types [1], while incineration may produce harmful substances such as dioxins during a high-temperature process that can accumulate in the environment and food chain [3]. Cotton fiber is one of the most common fibers for textiles [4]. Cotton fabrics have good moisture absorbency and heat isolation but also have poor wrinkle resistance and elasticity [5]. Therefore, synthetic polymeric fibers are usually mixed with cotton fibers to obtain proper garment properties [6]. Among them, elastane (also known as spandex) is a typical synthetic elastic fiber that exhibits a highly reversible extension of 400–800% [7]. Due to its high elasticity, elastane has become the prerequisite for fashionable or functional apparel such as outer clothing, leisurewear, underwear, and sportswear, and it has a 30–40% growth in worldwide consumption per year [8]. In 2010, an estimated 80% of clothing sold in the United States contained elastane [9]. In chemical terms, elastane is a synthetic macromolecule with at least 85% of segmented thermoplastic polyurethane (TPU) [7], in which a hard segment contains diisocyanate and short diol chain extender residues responsible for the stiffness and toughness and a soft segment contains polyols such as polyether and polyester responsible for the resilience, extensibility, and flexibility of polyurethane [10]. Recent studies focused on the recycling of multi-material textiles such as cotton–polyester blends [11], viscose–polyester blends [12], wool–polyester blends [13], nylon–cotton blends [14], and nylon–elastane blends [15]. However, the research on the blend of cotton and elastane fibers has been seldom reported. Moreover, many studies involved at least two steps, separation and reuse; for example, Navone et al. [13] performed enzymatic digestion of wool fibers to recover polyester fibers from wool–polyester blends. It will be more convenient and cost-effective if the blended fibers can be recycled in one treatment.

Cellulose is one of the most important natural biopolymers, which can be used in various fields [16,17]. Dissolution and regeneration of waste cotton fabrics is a convenient way to fabricate cellulose films. Several solvent systems have been reported to dissolve waste cotton fibers, such as LiOH/urea/H_2_O, NaOH/urea/H_2_O, phosphoric acid/H_2_O, H_2_SO_4_/H_2_O, lithium chloride (LiCl)/*N*,*N*-dimethylacetamide (DMAc), organic electrolyte solution/ionic liquid system, and ionic liquids [18,19,20,21,22,23,24]. Among them, H_2_SO_4_ aqueous solution is easy to operate and can rapidly dissolve cellulose at low temperature [25]; NaOH/urea aqueous solvent has been reported as an effective and promising system to dissolve cellulose when precooled to −12.0 °C [26]; LiCl/DMAc is also a well-known cellulose solvent with high stability [27]. Currently, regenerated cellulose (RC) films have been extensively studied for various potential applications in biomedicine [27], packaging [28], thermal management [29], energy storage [30], and optical/electronic devices [31].

In our previous work, three typical waste cotton fabrics (t-shirts, bedsheets, and jeans) were successfully recycled into regenerated cellulose films [24]. Therefore, in this study, we aim to further investigate the dissolution and regeneration of waste cotton–elastane fabrics and study the effect of elastane fibers on the structure and properties of regenerated cellulose films. T-shirts with 95% cotton and 5% elastane were selected to be dissolved in H_2_SO_4_ aqueous solution, NaOH/urea aqueous solution, or LiCl/DMAc solution. Regenerated cellulose films were obtained from these three solvent systems, and their structures were studied by Fourier transform infrared spectroscopy (FT-IR) and scanning electron microscope (SEM). In addition, the properties of regenerated cellulose films, including transparency, mechanical properties, thermal stability, and water vapor permeability (WVP), were investigated.

## 2. Materials and Methods

### 2.1. Materials

Worn T-shirts (95% cotton/5% elastane) were selected for this research. Sulfuric acid (95.0–98.0%) and sodium hydroxide (≥97.0%) were purchased from Sigma-Aldrich (Oakville, ON, Canada). Acetone (≥99.5%), lithium chloride (≥99.7%), *N*,*N*-dimethylacetamide (≥99%), *N*,*N*-dimethylformamide (DMF, ≥99.8%), and urea (≥99.6%) were purchased from Fisher Scientific (Ottawa, ON, Canada).

### 2.2. Waste Textile Dissolution and Regeneration

The dissolution and regeneration of waste cotton–elastane fabric were carried out according to our previous work with some modifications [24], and the dissolution conditions in different solvent systems are summarized in Table 1. Briefly, the textile was ground (KRUPS, Toronto, ON, Canada) until no fabric pieces could be observed, and the pre-treatment was required for the dissolution in H_2_SO_4_ and NaOH/urea aqueous solvents. A certain amount of fibers was dissolved with mechanical stirring (IKA Eurostar 60 digital mixer, Wilmington, NC, USA). The obtained solutions were degassed by centrifugation (Eppendorf centrifuge 5430, NRW, Hamburg, Germany) at 3000 rpm for 8 min and then spread on a glass plate and regenerated in coagulation baths. The cellulose films prepared from H_2_SO_4_ aqueous solvent, NaOH/urea aqueous solvent, and LiCl/DMAc solvent were washed thoroughly with water; dried in air at room temperature; and coded as HRC, NRC, and DRC, respectively.

### 2.3. Characterization

#### 2.3.1. Elastane Fiber Dissolution in Three Solvent Systems

In order to investigate the status of elastane fibers in three solvent systems, the obtained solutions after mechanical stirring were centrifuged at 7830 rpm for 30 min, and the precipitates were separated, washed with water, and freeze-dried (FreeZone Console Freeze dryer 12L, Labconco, Kansas City, MO, USA). They were then characterized by FT-IR spectrometer (Cary 630, Agilent Technology, Mississauga, ON, Canada), and the spectra were recorded as the average of 72 scans with 2 cm^−1^ resolution in the 4000–650 cm^−1^ region. The precipitates were also observed by an optical microscope (LMC-1000 Series, Laxco, Mill Creek, WA, USA) equipped with a SeBaCam digital camera (Laxco, Mill Creek, WA, USA).

#### 2.3.2. Regenerated Cellulose Film Structure

The structure of regenerated cellulose films was investigated using an FT-IR spectrometer following the same procedure. The morphology of the films with and without DMF treatment was observed by using a Hitachi TM1000 SEM (Schaumburg, IL, USA) at an acceleration voltage of 4 kV. The film samples were sputtered with 4 nm gold–platinum by a Leica EM ACE200 low vacuum coater (Vaughan, ON, Canada) prior to observation and photographing. The films were either directly observed or immersed in DMF for 12 h at room temperature to remove any remaining elastane component and then washed and dried for the observation.

#### 2.3.3. Regenerated Cellulose Film Properties

The mechanical properties of regenerated cellulose films (50 mm × 10 mm) were tested on an eXpert 7601 single-column testing machine (ADMET, Norwood, MA, USA) at 25 °C according to standard ASTM D882. The initial grip separation distance was set as 20 mm with a separation speed of 5 mm/min. Three strips with the dimensions of 5 cm × 1 cm (length × width) were cut from each film, and the thickness was determined from SEM cross-section images. All the films were stored and tested at 25 °C and 50% RH. A modified cup method was used to evaluate the WVP of regenerated cellulose films according to ASTM E96-92 standard [32]. The films were sealed in glass jars containing anhydrous calcium chloride. Then, the jars were placed in a desiccator containing distilled water at 25 °C. The weights of the jars were measured periodically, and the WVP of films was calculated using the following equation [32]:

(1) $$WVP=\frac{\Delta m\times k}{A\times \Delta T\times \Delta P}$$

where Δ*m* is the weight change of the jar (g), *k* is the film thickness (m), *A* is the exposed area of the film, Δ*T* represents the time, and Δ*P* is the partial pressure difference that existed between the two sides of the film (Pa).

Thermogravimetric analysis (TGA) of the regenerated cellulose films was performed using a thermogravimetric analyzer Q500 (TA instruments, New Castle, DE, USA) with a heating rate of 10 °C/min in nitrogen (40 mL/min) from 50 to 600 °C [33]. Thermograms of samples were collected, and the OriginPro software was used for calculating the first derivatives of thermograms (DTG) and the percentage weight loss.

### 2.4. Statistical Analysis

The experiments were carried out in triplicate, and the results were represented as the mean ± standard deviation. Statistical evaluation was carried out by analysis of variance (ANOVA) followed by multiple comparison tests using Duncan’s multiple range test. All of the analyses were carried out through SPSS statistical software (IBM, Armonk, NY, USA) with significant differences within samples at *p* < 0.05.

## 3. Results and Discussion

### 3.1. Dissolution of Cotton–Elastane Fabric in Three Solvent Systems

**Figure 1:**
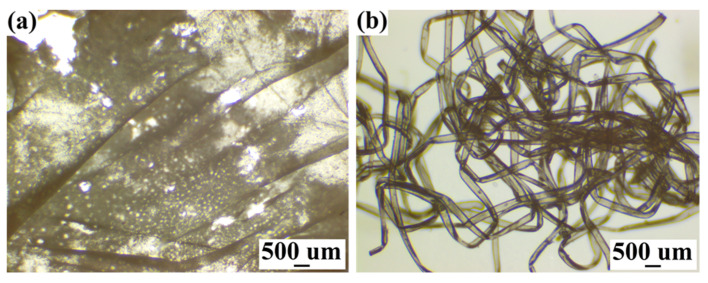
Optical microscopic images of undissolved components separated from (**a**) H_2_SO_4_ aqueous solvent system and (**b**) NaOH/urea aqueous solvent system.

The waste cotton textile containing elastane fibers was dissolved in three solvent systems. Since the capacity of these solvent systems varies, different pre-treatment conditions (sulfuric acid hydrolysis) were introduced to facilitate the dissolution process. The existence of elastane fibers did not show any significant effect on the dissolution of waste cotton fabrics. Similar to our previous work [24], both H_2_SO_4_ and NaOH/urea aqueous solvent systems required the pre-treatment of acid hydrolysis, while LiCl/DMAc solvent system could directly dissolve the sample after multiple activation steps (e.g., swelled in water and washed with acetone and DMAc). No obvious elastane fibers or aggregates could be distinguished in all three cellulose solutions, and the fabric concentrations in H_2_SO_4_ and NaOH/urea systems were higher than those in LiCl/DMAc solvent. To better understand the effect of solvent systems on the elastane fibers, the undissolved components were centrifuged out from three cellulose solutions and observed with an optical microscope. As shown in Figure 1, some plastic flakes were obtained from H_2_SO_4_ solution, which might be the partially hydrolyzed elastane under strong acidic condition [34]. It is worth noting that the elastane fibers were well retained in NaOH/urea solvent system but completely dissolved in LiCl/DMAc solution [35].

### 3.2. Structure of Regenerated Cellulose Films

**Figure 2:**
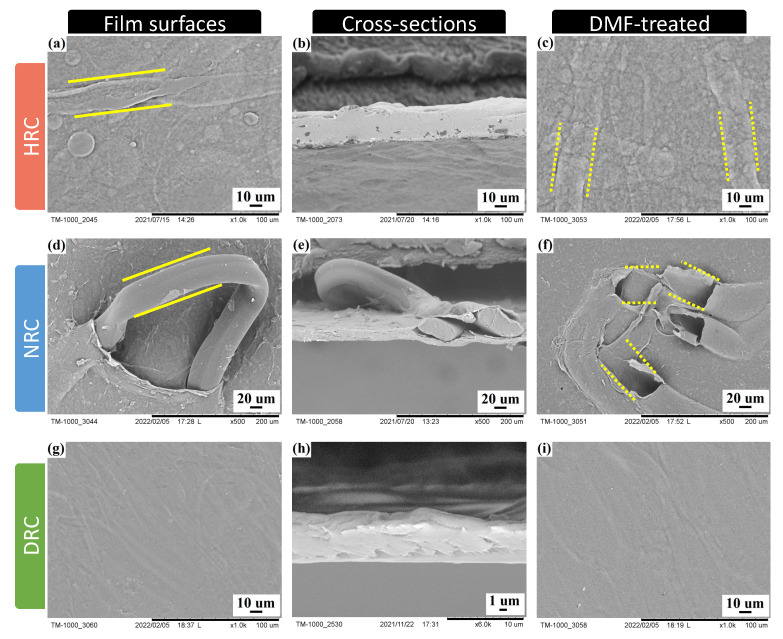
SEM images of regenerated cellulose film surfaces (left), cross-sections (middle), and DMF-treated film surfaces (right) prepared from three solvent systems: (**a**–**c**) HRC, (**d**–**f**) NRC, and (**g**–**i**) DRC.

In order to examine the morphology of regenerated cellulose films prepared from three solvent systems, the SEM images were collected to observe the surfaces and cross-sections of regenerated cellulose films. As shown in Figure 2, partial fibers appeared on the surface of HRC film and intact fibers existed on NRC film. Since these components were successfully removed using DMF solvent (highlighted in Figure 2c,f), it was confirmed that they were undissolved elastane fibers and were in accordance with Figure 1. In addition, no fibers were found on the surface of the DRC film, and the DMF treatment did not cause any obvious change, which indicated the dissolution of both cotton and elastane fibers by LiCl/DMAc solvent system. Among three regenerated cellulose films, DRC film possessed a relatively smooth and uniform structure with few ripple-like grains that might be contributed by the rapid shrinkage of film during the coagulation process. To be noticed, the surface and inner structure of NRC film were disturbed by the well-retained elastane fibers, which might affect the properties of regenerated cellulose films.

**Figure 3:**
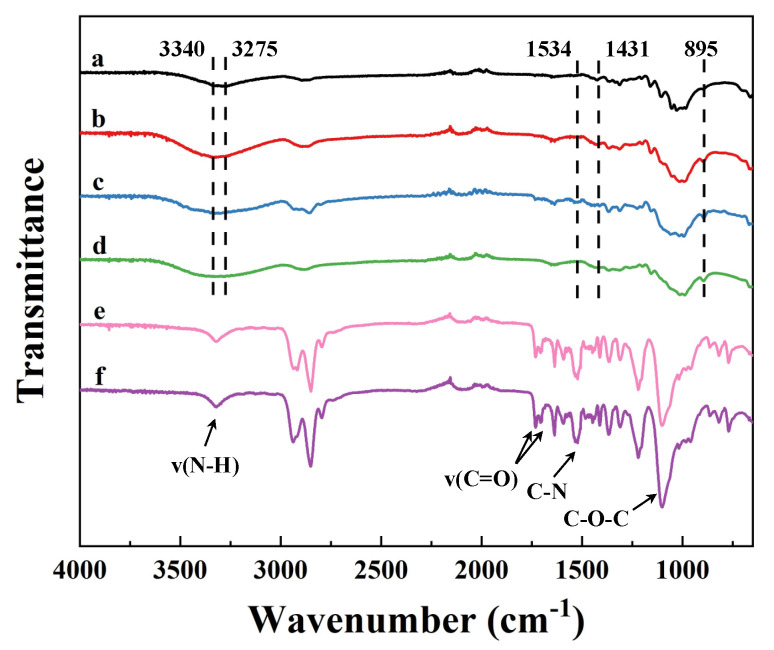
FT-IR spectra of (**a**) cotton–elastane fabrics, (**b**) HRC, (**c**) NRC, (**d**) DRC, and undissolved components separated from (**e**) H_2_SO_4_ solvent system and (**f**) NaOH/urea solvent system.

To further investigate the effect of dissolution and the structure of regenerated cellulose films, FT-IR spectra of waste textile, regenerated cellulose films, and undissolved components were collected. As shown in Figure 3a, two peaks of waste textile were observed at 3275 cm^−1^ and 3340 cm^−1^ representing the intramolecular hydrogen bonds in cellulose I structure [24]. The peaks at 1431, 1364, 1333, and 1314 cm^−1^ were attributed to CH_2_ bending, CH bending, OH bending, and CH_2_ rocking vibration, respectively [36]. However, no characteristic peaks of elastane could be found on the spectrum of waste textile, which might be due to the low amount of elastane fibers in the fabric. The change in cellulose crystalline structures after dissolution and regeneration was noticed from the reduction or even disappearance of the characteristic band at 1431 cm^−1^ (Figure 3b–d) [36]. In addition, the band at 895 cm^−1^, representing the amorphous cellulose [37], showed an increase in its intensity. Thus, the band intensity ratio I_1430_/I_900_ decreased, suggesting that the crystalline structure of cellulose changed from cellulose I to cellulose II [37]. Only NCR film showed a characteristic peak of TPU at 1534 cm^−1^ representing the C-N stretching of amide II [38]. This was because the elastane fibers were retained and exposed on the surface of the NCR film. The undissolved components were also analyzed and are shown in Figure 3e,f. In both spectra, a weak peak at 3328 cm^−1^ was attributed to the hydrogen bonds between N-H groups and ester groups (C=O) [39]. Two bands at 1731 cm^−1^ and 1707 cm^−1^ were related to the stretching of free and bonded C=O groups [40]. A characteristic peak of amide II of TPU was found at 1534 cm^−1^, and a strong band of C-O-C stretching occurred at 1101 cm^−1^. Therefore, it was confirmed that the undissolved components were mainly elastane components [38].

### 3.3. Properties of Regenerated Cellulose Films

**Figure 4:**
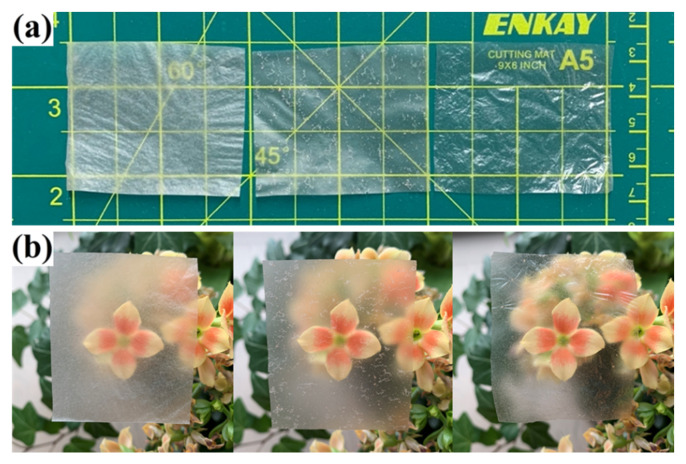
Digital pictures of regenerated cellulose films (HRC, NRC, and DRC from left to right) under (**a**) room light and (**b**) natural light.

It is evident in Figure 4 that all regenerated cellulose films were translucent under room light and natural light. In general, the transparency of regenerated cellulose films is affected by the content of cellulose and the presence of undissolved particles [24]. It has been reported that higher cellulose content could lead to a more compact structure [41]. In addition, undissolved particles can cause optical scattering and internal reflection of light resulting in a decrease in transparency. The DRC film showed better transparency because the content of cellulose in the LiCl/DMAc solvent system was low and the elastane fibers were completely dissolved. However, undissolved elastane components were present in HRC and NRC films and increased the optical scattering.

**Figure 5:**
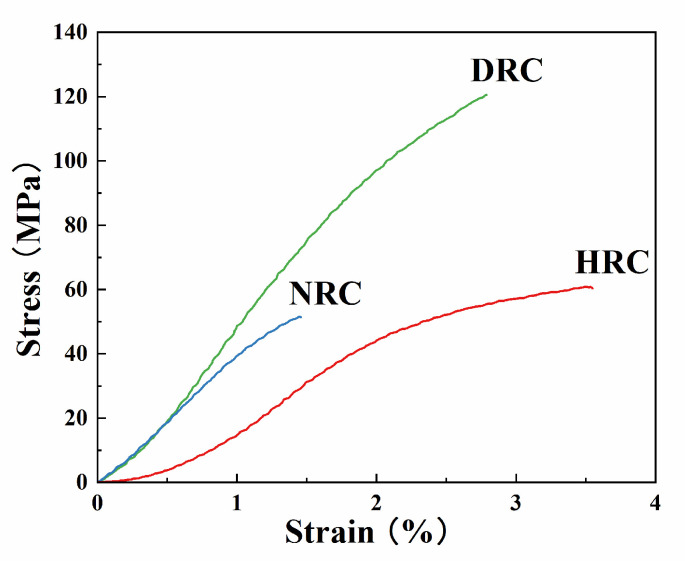
Stress–strain curves of regenerated cellulose films from three solvent systems.

Mechanical properties of regenerated cellulose films are summarized in Table 2, and the typical stress–strain curves are shown in Figure 5. NRC films exhibited the lowest tensile strength, Young’s modulus, and elongation at break among three samples. This might be due to the existence of large undissolved elastane fibers that interrupted the continuity of cellulose film. However, the tensile strengths of HRC and NRC were slightly higher than those of our former regenerated cellulose films from pure cotton textile, while the DRC film was three times stronger than the sample without elastane fibers [24]. This indicated that the elastane component did not have any negative effect on the mechanical properties of regenerated cellulose films, and the dissolved elastane fibers could even reinforce the film by forming a composite structure [42]. WVP values of regenerated cellulose films are also listed in Table 2. The DRC film exhibited the best water vapor barrier property (1.03 ± 0.53 × 10^−7^ g m^−1^ h^−1^ Pa^−1^), which was significantly lower than those of HRC and NRC films (*p* < 0.05). This could be due to the dissolved elastane component in DRC film. As shown in Table 2, the tensile strength and WVP values of the obtained films were comparable with those of RC films prepared from corncob residue [43], pure cotton textile [24], bamboo [44], and cotton linter [45,46], suggesting that waste cotton–elastane fabric is also a promising source for the preparation of RC films.

**Figure 6:**
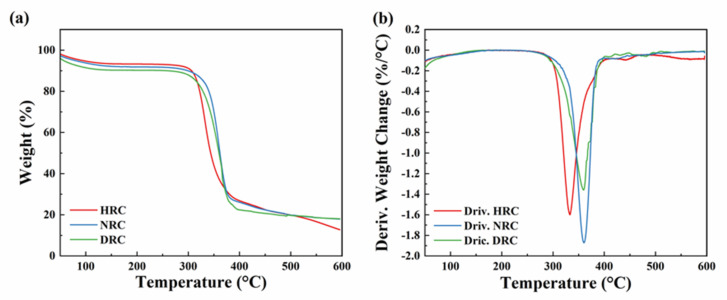
(**a**) Thermogravimetric analysis and (**b**) derivatives of thermogram curves of regenerated cellulose films prepared from three solvent systems.

TGA and DTG curves are depicted in Figure 6. The initial weight loss at about 100 °C was due to the dehydration of regenerated films [47]. The main weight loss occurred in the range of 300–390 °C, corresponding to cellulose thermal decomposition and carbonization [33] and urethane hard segment degradation [48]. The maximum decomposition temperatures (T_max_) of HRC, NRC, and DRC films were 332.68, 360.26, and 359.97 °C, respectively. Among them, the NRC and DRC films exhibited relatively higher T_max_ values, which might be attributed to the higher cellulose concentration in NaOH/urea solution [40] and the dissolution of elastane fibers in LiCl/DMAc solvent [49].

## 4. Conclusions

In this work, waste textile containing elastane fibers was successfully converted into regenerated films using H_2_SO_4_ aqueous solvent, NaOH/urea aqueous solvent, and LiCl/DMAc solvent. All three solvent systems could effectively dissolve the cellulose fibers, while resulting in different forms of elastane components. The NRC film contained large undissolved elastane fibers, and its mechanical properties were weaker than the other two samples. The elastane fibers were partially hydrolyzed in H_2_SO_4_ solvent but were completely dissolved in LiCl/DMAc solvent, so the DRC film had the uniform structure, high transparency, improved tensile strength of 121.63 ± 6.16 MPa, lowest WVP of 1.03 ± 0.53 × 10^−7^ g m^−1^ h^−1^ Pa^−1^, and a maximum decomposition temperature of 359.97 °C. Therefore, it is demonstrated that the waste cotton–elastane fabrics can be recycled by the three solvent systems investigated in this work, which provides a promising strategy to relieve the environmental pressure caused by waste textiles.

## Figures and Tables

**Table 1:** Dissolution conditions of waste cotton–elastane fabric in three solvent systems.

Regenerated Film	Pre-Treatment	Solvents	Dissolution Time	Dissolution Temperature (°C)	Fabric Concentration (wt%)
HRC	48 h in 20% (*w*/*v*) H_2_SO_4_	64% (*w*/*v*) H_2_SO_4_	45 min	0	3
NRC	60 h in 30% (*w*/*v*) H_2_SO_4_	7% NaOH/12% Urea	45 min	0	4
DRC	/	8% LiCl/92% DMAc	3–5 days	80	0.7

**Table 2:** Mechanical properties and water vapor permeability of regenerated cellulose films from various raw materials. Different letters in the same columns indicate the significant difference within this work (*p* < 0.05).

Sample	Raw Material	Solvent Used	Tensile Strength (MPa)	Elongation at Break (%)	Young’s Modulus (MPa)	WVP (g m^−1^ h^−1^ Pa^−1^)	Ref.
HRC	Cotton–elastane textile	64% H_2_SO_4_	67.30 ± 2.82 ^b^	3.70 ± 0.72 ^a^	3415.56 ± 89.35 ^b^	3.50 ± 0.57 × 10^−7 a^	This work
NRC	Cotton–elastane textile	NaOH/urea	51.00 ± 1.92 ^c^	1.49 ± 0.15 ^b^	3223.08 ± 314.45 ^b^	2.66 ± 0.32 × 10^−7 b^	This work
DRC	Cotton–elastane textile	LiCl/DMAc	121.63 ± 6.16 ^a^	2.97 ± 0.14 ^a^	5746.35 ± 343.84 ^a^	1.03 ± 0.53 × 10^−7 c^	This work
RC	Corncob residue	Ionic liquid	70.63	~9.00	-	9.07 × 10^−8^	[43]
RC	Bamboo	LiCl/DMAc	81.09	10.98	360	-	[44]
RC	Cotton textile	NaOH/urea	76.21	~3.50	~3250	8.10 × 10^−8^	[24]
RC	Cotton linter	NaOH/urea	73.40	5.10	5500	8.21 × 10^−6^	[45]
RC	Cotton linter	NaOH/urea	48.34	~2.00	38,600	6.16 × 10^−7^	[46]

## Data Availability

The data presented in this study are available on request from the corresponding author.
